# Lung vasodilatory response to inhaled iloprost in experimental pulmonary hypertension: amplification by different type phosphodiesterase inhibitors

**DOI:** 10.1186/1465-9921-6-76

**Published:** 2005-07-20

**Authors:** Ralph Theo Schermuly, Christiane Inholte, Hossein Ardeschir Ghofrani, Henning Gall, Norbert Weissmann, Andreas Weidenbach, Werner Seeger, Friedrich Grimminger

**Affiliations:** 1 Medical Clinic II/V, Justus-Liebig-University Giessen, 35392 Giessen, Germany

## Abstract

Inhaled prostanoids and phosphodiesterase (PDE) inhibitors have been suggested for treatment of severe pulmonary hypertension. In catheterized rabbits with acute pulmonary hypertension induced by continuous infusion of the stable thromboxane analogue U46619, we asked whether sildenafil (PDE1/5/6 inhibitor), motapizone (PDE3 inhibitor) or 8-Methoxymethyl-IBMX (PDE1 inhibitor) synergize with inhaled iloprost. Inhalation of iloprost caused a transient pulmonary artery pressure decline, levelling off within <20 min, without significant changes in blood gases or systemic hemodynamics. Infusion of 8-Methoxymethyl-IBMX, motapizone and sildenafil caused each a dose-dependent decrease in pulmonary artery pressure, with sildenafil possessing the highest efficacy and at the same time selectivity for the pulmonary circulation. When combining a *per se *ineffective dose of each PDE inhibitor (200 μg/kg × min 8-Methoxymethyl-IBMX, 1 μg/kg × min sildenafil, 5 μg/kg × min motapizone) with subsequent iloprost nebulization, marked amplification of the prostanoid induced pulmonary vasodilatory response was noted and the area under the curve of P_PA _reduction was nearly threefold increased with all approaches, as compared to sole iloprost administration. Further amplification was achieved with the combination of inhaled iloprost with sildenafil plus motapizone, but not with sildenafil plus 8MM-IBMX. Systemic hemodynamics and gas exchange were not altered for all combinations. We conclude that co-administration of minute systemic doses of selective PDE inhibitors with inhaled iloprost markedly enhances and prolongs the pulmonary vasodilatory response to inhaled iloprost, with maintenance of pulmonary selectivity and ventilation perfusion matching. The prominent effect of sildenafil may be operative via both PDE1 and PDE5, and is further enhanced by co-application of a PDE3 inhibitor.

## Introduction

Severe pulmonary hypertension is a fatal disease with short life expectancy [1,2]. Continuous intravenous administration of prostacyclin was documented to improve exercise capacity and survival in patients with idiopathic pulmonary arterial hypertension (IPAH, formerly primary pulmonary hypertension, PPH) [1,3]. Possible disadvantages of this approach are catheter related septic events and systemic side effects including serious systemic hypotension. In patients with pulmonary hypertension associated with pulmonary fibrosis, systemic administration of vasodilators results in ventilation perfusion mismatch and impairment of arterial oxygenation. Inhalation of aerosolized iloprost, a long-acting prostacyclin analogue, has been shown to cause selective pulmonary vasodilatation in both primary and secondary pulmonary hypertension [4-6]. Long term use of nebulized iloprost was described to improve exercise capacity, event-free survival and hemodynamics in severe IPAH, and this finding was supported by a randomized, controlled phase III study in patients in NYHA class III and IV [7].

The use of aerosolized iloprost for acute pulmonary vasodilatation and putative long-term anti-remodeling effects in severe chronic pulmonary hypertension does, however, demand 6 – 12 inhalations per day, as the vasodilatory effect levels off within ~60 min post nebulization. Against this background, recent studies addressed the impact of selective phosphodiesterase (PDE) inhibitors on prostacyclin-induced acute pulmonary vasodilatation, reporting a marked amplification and prolongation of the vasodilatory response to inhaled PGI_2 _[8]. PDEs are enzymes that inactivate cyclic AMP and cyclic GMP, the second messengers of prostacyclin and NO [9,10]. The characterization of the various PDEs currently known has largely profited from the employment of selective PDE inhibitors. Concerning the lung vasculature, the presence of the PDE isoenzymes 1, 3, 4 and 5 in the cytosolic and particulate phases (homogenized human pulmonary artery tissue) has been demonstrated [11].

Phosphodiesterase 1 is Ca^2+^/calmodulin dependant and hydrolyzes both cGMP and cAMP. PDE3 does possess high affinity for both cAMP and cGMP, with V_max _for cAMP usually greater than that for cGMP [9,12]. PDE4 enzymes are characterized by their high affinity to cAMP, with cGMP representing a very poor substrate. In contrast, PDE 5 is cGMP-specific and was found to be highly expressed in lung tissue [13,14].

Recent clinical data suggest that the PDE 1/5/6 inhibitor sildenafil (IC_50 _values 280 nM, 3.5 nM and 37 nM, respectively [15]), which has been approved for the treatment of erectile dysfunction, is an effective pulmonary vasodilator in patients with pulmonary arterial hypertension [16-21]. Based on a very recent positive phase III study, sildenafil has been approved for the treatment of pulmonary hypertension in US. Interestingly, it has been shown that sildenafil synergizes with inhaled iloprost in patients with pulmonary hypertension [16,22]. Hitherto no attempt was undertaken to clarify, which of the PDEs addressed by sildenafil is the most relevant for the effect of this agent in the pulmonary circulation, and whether combinations with further selective PDE inhibitors might even enhance the sildenafil effect. To address this issue, systemic application of *per se *ineffective doses of specific PDE inhibitors in companion with inhalation of iloprost was undertaken in an experimental model of pulmonary hypertension in the present study.

## Methods

### Materials

8-Methoxymethyl-IBMX (8-Methoxymethyl-3-isobutyl-1-methylxanthine) and the thromboxane-A_2 _mimetic U46619 were supplied by Sigma (Deishofen, Germany). Sildenafil was obtained from Pfizer (Sandwich, UK) and iloprost (Ilomedin^®^) was obtained from Schering A.G. (Berlin, Germany). All other chemicals and drug supplies were from standard commercial sources.

### Surgical Preparation

New zealand white rabbits weighing between 2.8 and 3.1 kg of either sex were anesthetized with a mixture of xylazine and ketamine and anticoagulated with 200 U/kg heparin [8]. Anaesthesia was maintained by a constant intravenous infusion of xylazine and ketamine through the right peripheral ear vein. Animals were tracheostomized and ventilated using a volume-controlled respirator (cat ventilator, Hugo Sachs Elektronik, March Hugstetten, Germany) with 8 ml/kg bodyweight and a frequency of 40 min^-1^. FiO_2 _was set at 0.5 and a positive endexpiratory pressure of 0.5 mmHg was used throughout. The left A. carotis was cannulated for arterial pressure monitoring and a pulmonary artery catheter (4 Fr, Braun, Melsungen, Germany) was inserted into the pulmonary artery through the right external jugular vein.

### Hemodynamics and blood gases

Mean pulmonary artery pressure (P_PA_) and mean aortic pressure (P_SA_) were continuously recorded with fluid-filled force transducers (Braun, Combitrans, FRG). The level of the left atrium was set to zero. As described previously, pulmonary artery occlusion pressure was measured by gentle forwarding of the catheter to wedge position [23]. Pulmonary and systemic vascular resistances were calculated by standard formulas. As described previously, cardiac output (CO) was calculated by using the Fick principle [8]. Briefly, arterial and venous blood samples (1 ml) were stored on ice, and hemoglobin and oxygen saturation were measured using an OSM2 Hemoximeter (Radiometer-Copenhagen, Denmark). Oxygen uptake of the animals was measured online (Labotect O_2_-Controller, Goettingen, Germany).

### Nebulization

Iloprost was nebulized by means of an ultrasonic nebulizer (Pulmo Sonic 5500, DeVilbiss Medizinische Produkte GmbH, Langen, Germany) which produces an aerosol with a mass median aerodynamic diameter (MMAD) of 4.5 μm and a geometric standard deviation (GSD) of 2.3. The nebulizer was placed in the inspiratory limb of the ventilation system as described previously [24].

### Experimental protocols

U46619 was continuously infused (dose range 0.5 to 2 μg/kg min) to increase pulmonary artery pressure from ~16 at baseline to ~26 mmHg within 20 min. As described previously, stable pulmonary hypertension is established by this approach [8]. Dose-effect curves of intravenous sildenafil, motapizone and 8MM-IBMX were established after reaching a stable pressure plateau, performing short-term infusions (10 min) with randomized doses of these agents. Hemodynamics and blood gases were measured at the end of the 10 min infusion period. A total dose of 0.4 ± 0.08 μg/kg iloprost, nebulized within a 10 min aerosolization maneuver, was used throughout all studies with iloprost inhalation. In the group with sole administration of this inhalative agent, the nebulization was performed after reaching a stable pressure plateau. In the combination experiments, the PDE inhibitors were administered intravenously at a dose which by itself did not reduce PAP significantly as short-term infusion (10 min), and iloprost was nebulized subsequently.

### Data analysis

All data are given as means ± SEM. Differences between the different groups were assessed by use of analysis of variance and Student-Newman-Keuls test for multiple comparisons with a p value < 0.05 regarded to be significant.

## Results

### Baseline and U46619-induced pulmonary hypertension

The continuous infusion of 1.3 ± 0.9 μg/kg min U46619 resulted in a significant increase of pulmonary artery pressure (P_PA_) to 26 mmHg as compared to 16 mmHg prior to U46619 (Table 1). Cardiac output and mean systemic pressure (P_SA_) did not change significantly. The pulmonary vascular resistance increased from 275 to 592 dyne/s cm^-5 ^m^2^. No significant changes in blood gases were measured as compared to baseline values.

**Table 1:** Summarized data of hemodynamics and blood gases in rabbits with U46619-induced pulmonary hypertension and inhalation of iloprost in the absence and presence of sub-threshold intravenous PDE inhibitors.^Δ^

	**Control/U46619**		**U46619/Ilo**		**U46619/Ilo/Sil**		**U46619/Ilo/8MM-IBMX**		**U46619/Ilo/Mota**		**U46619/Ilo/8MM-IBMX/Sil**		**U46619/Ilo/Mota/Sil**	
	**Pre**	**post**	**pre**	**post**	**pre**	**post**	**pre**	**post**	**pre**	**post**	**pre**	**post**	**pre**	**post**
**P_SA_, mmHg**	111 ± 4	105 ± 3	111 ± 3	111 ± 3	106 ± 3	105 ± 2	107 ± 2	104 ± 4	100 ± 3	96 ± 3	99 ± 3	97 ± 2	93 ± 4	92 ± 4
**P_PA_, mmHg**	15.9 ± 0.3	25.9* ± 0.4	25.8 ± 0.6	22.7* ± 0.4	25.7 ± 0.2	21.8* ± 0.3	24.8 ± 0.7	21.1* ± 0.8	27.3 ± 1,1	21.0* ± 0.6	25.0 ± 0.4	20.4* ± 0.3	24.7 ± 0.4	19.3* ± 0.4
**CO, ml/min**	555 ± 23	544 ± 38	432 ± 27	452 ± 25	444 ± 19	488 ± 35	393 ± 24	437 ± 29	417 ± 21	446 ± 15	454 ± 27	505 ± 30	501 ± 35	555 ± 52
**PAOP, mmHg**	7.2 ± 1.2	7.6 ± 0.8	8.0 ± 1.1	7.3 ± 1.3	7.7 ± 0.9	7.4 ± 1.1	7.7 ± 0.8	7.6 ± 1.2	6.9 ± 1.3	7.3 ± 1.1	7.7 ± 1.2	7.7 ± 1.1	7.8 ± 0.8	7.2 ± 1.1
**PVRI, dyne/s cm^-5 ^m^2^**	275 ± 34	592* ± 49	725 ± 39	599* ± 37	713 ± 58	519* ± 44	765 ± 67	543* ± 63	861 ± 64	540* ± 71	670 ± 75	442* ± 52	593 ± 56	383* ± 61
**P_a_O_2_, mmHg**	226 ± 17	199 ± 12	183 ± 12	177 ± 10	191 ± 6	203 ± 4	228 ± 7	188 ± 12	201 ± 7	197 ± 11	171 ± 10	173 ± 11	179 ± 10	174 ± 14
**PH_a_**	7.42 ± 0.02	7.35 ± 0.02	7.37 ± 0.03	7.32 ± 0.01	7.36 ± 0.02	7.32 ± 0.02	7.41 ± 0.02	7.38 ± 0.02	7.33 ± 0.01	7.31 ± 0.01	7.35 ± 0.02	7.34 ± 0.02	7.33 ± 0.02	7.29 ± 0.01
**P_a_CO_2 _mmHg**	42.7 ± 1.3	43.0 ± 3.5	41.5 ± 2.0	40.7 ± 1.0	42.0 ± 4.1	45.2 ± 4.0	36.2 ± 1.6	37.7 ± 1.5	44.3 ± 1.2	46.2 ± 1.2	45.9 ± 1.7	47.6 ± 1.4	36.9 ± 2.0	38.0 ± 1.8
**P_v_O_2_, mmHg**	46.7 ± 1.3	44.1 ± 2.4	39.9 ± 1.7	42.6 ± 1.2	39.7 ± 2.3	44.9 ± 1.4	35.8 ± 1.5	39.7 ± 2.1	40.7 ± 1.64	42.7 ± 1.2	39.7 ± 2.5	44.9 ± 2.6	41.8 ± 1.2	44.0 ± 1.6
**PH_v_**	7.30 ± 0.01	7.24 ± 0.02	7.34 ± 0.03	7.29 ± 0.01	7.23 ± 0.02	7.20 ± 0.02	7.37 ± 0.02	7.31 ± 0.03	7.28 ± 0.01	7.26 ± 0.01	7.29 ± 0.02	7.28 ± 0.02	7.28 ± 0.02	7.24 ± 0.01
**P_v_CO_2_, mmHg**	49.8 ± 0.9	54.9 ± 1.2	48.9 ± 2.3	49.7 ± 1.1	59.2 ± 1.6	60.6 ± 1.4	44.6 ± 2.5	44.9 ± 2.5	52.4 ± 1.4	54.2 ± 1.4	53.8 ± 2.0	52.9 ± 2.2	42.5 ± 2.3	45.9 ± 1.6

^Δ ^P_SA_, mean aortic pressure; P_PA_, mean pulmonary artery pressure; CO, cardiac output; PAOP, pulmonary artery occlusion pressure; PVR, pulmonary vascular resistance; P_a_O_2_, arterial PO_2_; pH_a_, arterial pH; P_a_CO_2_, arterial PCO_2_; P_v_O_2_, central venous PO_2_; pH_v_, central venous pH; P_v_CO_2_, central venous PCO_2_; Ilo, iloprost; Sil, sildenafil; 8MM-IBMX, 8-Methoxymethyl-3-isobutyl-1-methylxanthine; Mota, motapizone. The first two columns give summarized data for all groups. All other columns give pre- and post-iloprost nebulization data in animals undergoing preceding sub-threshold PDE-inhibitor infusion (n = 8 for each group). Asterisks indicate significant differences between pre and post-aerosolization values (* = p < 0.05).

### Dose-effect curves of PDE-inhibitors

**Figure 1:**
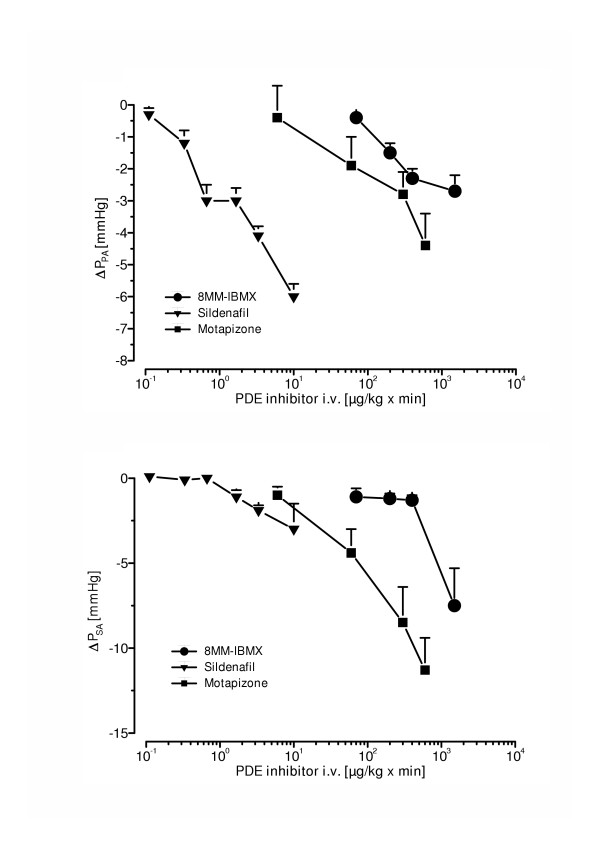
Dose effect curves of different PDE inhibitors on U46619-elicited pulmonary hypertension (a) and systemic arterial pressure (b). (a) Pulmonary artery pressure drop (ΔP_PA_, in mmHg) and systemic arterial pressure drop (ΔP_SA_, in mmHg) (b) are given (mean ± SEM of 6 independent experiments each). PDE-inhibitors were applied in different doses as short-term infusion.

Intravenous sildenafil, motapizone and 8MM-IBMX reduced P_PA _in a dose-dependent manner (Fig. 1A), with the dose-effect curves differing by ~two orders of magnitude between sildenafil and the two other compounds. As depicted in Fig. 1B, this pulmonary vasodilatation was accompanied by a significant systemic arterial pressure decrease in case of motapizone (dose range 6 – 600 μg/kg × min) and 8MM-IBMX (dose range 70 – 1500 μg/kg × min), but not in case of sildenafil (dose range 0.1 – 10 μg/kg × min).

### Nebulization of iloprost

**Figure 2:**
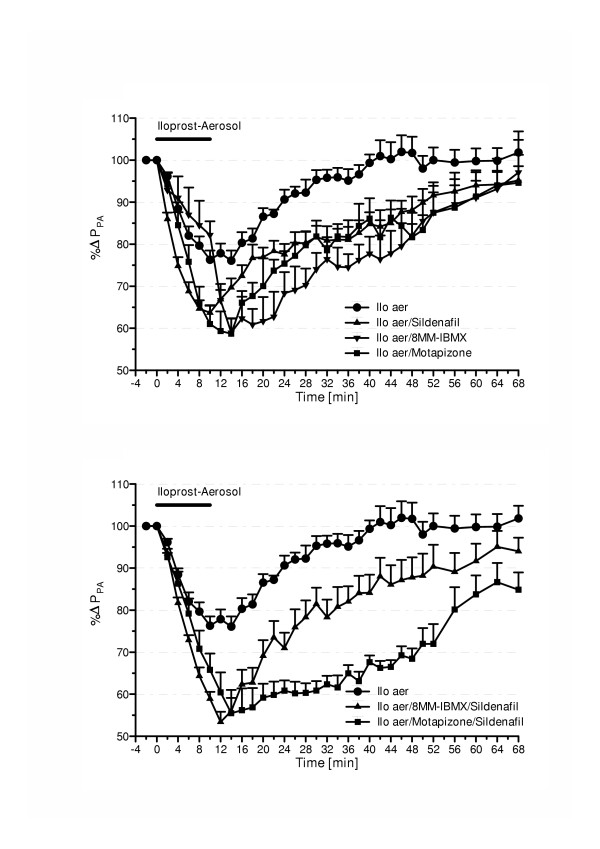
Influence of iloprost nebulization and its combination with sub-threshold doses of single (a) or combined (b) intravenous PDE inhibitors on U46619-elicted pulmonary hypertension. Pulmonary artery pressure (P_PA_, in % of U46619-induced increase) is given (mean ± SEM of 8 independent experiments each, SEM bars are missing when falling into symbol). Iloprost nebulization (Ilo aer; 0.4 μg/kg) is indicated by the horizontal bar. The PDE inhibitors were pre-applied as short-term infusion as follows: 200 μg/kg × min 8-Methoxymethyl-IBMX, 1 μg/kg × min sildenafil, 5 μg/kg × min motapizone.

**Figure 3:**
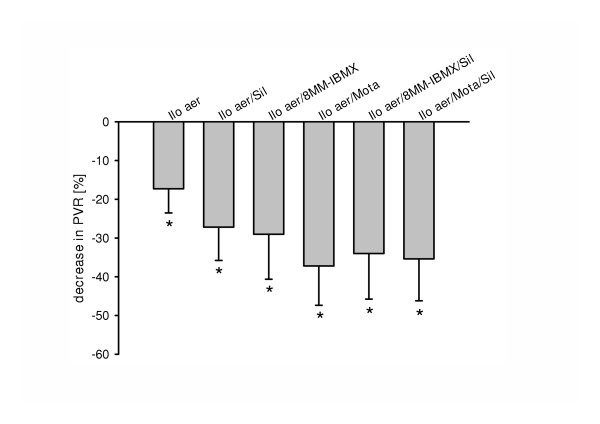
Influence of iloprost nebulization and its combination with sub-threshold doses of intravenous PDE inhibitors on U46619-elicted pulmonary hypertension. Decrease in pulmonary vascular resistance (PVR, in %) at the end of the nebulization period is given (mean ± SEM of 8 independent experiments each). The PDE inhibitors were pre-applied as short-term infusion as follows: 200 μg/kg × min 8-Methoxymethyl-IBMX, 1 μg/kg × min sildenafil, 5 μg/kg × min motapizone. *, p < 0.05 as compared to pre-nebulization value.

**Figure 4:**
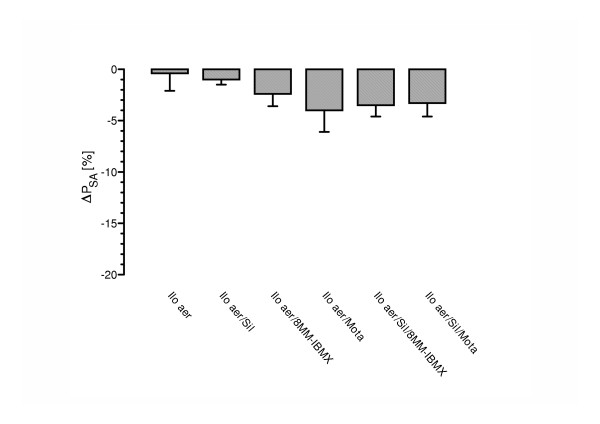
Influence of iloprost nebulization and its combination with sub-threshold doses of single or combined intravenous PDE inhibitors on systemic arterial pressure (P_SA_). The experiments correspond to those in Fig. 2; the decrease in P_SA _(in % of baseline) at the end of the iloprost nebulization (Ilo aer) period is given (mean ± SEM of 8 independent experiments each).

**Figure 5:**
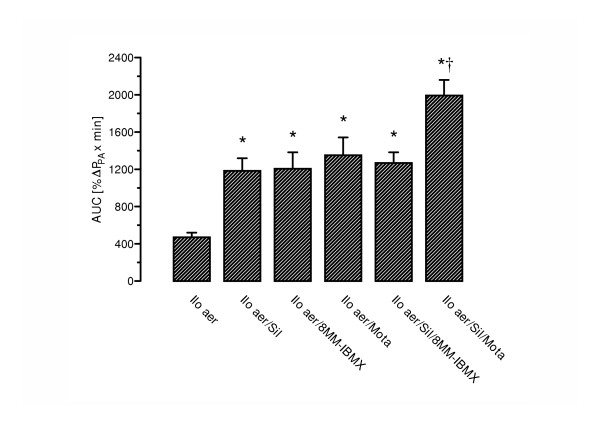
Influence of PDE inhibition on the area under the curve (AUC) of iloprost-induced decrease in pulmonary artery pressure (P_PA_). Measurements were performed from onset of iloprost nebulization (Ilo aer) until 68 min post aerosolization. AUC was calculated by standard techniques and is given as %ΔP_PA _× min. The maneuvers and dosages correspond to those depicted in Fig. 2. *, p < 0.05 as compared to sole iloprost nebulization; †, p < 0.05 as compared to all other groups.

Inhalation of 0.4 μg/kg aerosolized iloprost resulted in a significant decrease in U46619-induced pulmonary hypertension, from 25.8 ± 0.6 to 22.7 ± 0.4 mmHg P_PA _immediately after stop of nebulization (Table 1, Fig. 2a,b). Pulmonary vascular resistance decreased in response to the prostanoid by 18% (Fig. 3). No significant changes in blood gases, cardiac output and systemic arterial pressure were noted (Fig. 4). Within ~18 min, 95% of the U46619-induced P_PA _plateau was reached again. The calculated area under the curve (AUC) was 470 ± 49 %ΔP_PA _× min (Fig. 5).

### Combined administration of a per se ineffective intravenous PDE-inhibitors and iloprost nebulization

In the presence of sub-threshold motapizone (5 μg/kg × min), the iloprost-induced vasodilatation was significantly increased, with a maximum P_PA _drop of 40.6 ± 4.6% of the U46619-induced pressor response, as compared to 23.8 ± 2.2% for sole iloprost nebulization (Fig. 2a). In addition, motapizone enhanced the iloprost induced PVR reduction (37% versus 18%) (Fig. 3). Moreover, the duration of the vasodilatory response, defined by P_PA _values below 95% of the U46619-induced pressure plateau, was significantly prolonged, from 18 min to 50 min. The AUC was markedly increased to 1351 ± 192 %ΔP_PA _× min (p < 0.01; Fig. 5). Comparable efficacy was noted for intravenous sub-threshold sildenafil (1 μg/kg × min), which enhanced the maximum P_PA _drop to 36.3 ± 3.6% of the U46619-induced pressor response and PVR reduction to 27%, prolonged the post-nebulization vasodilatory effect to 50 min, and increased the AUC to 1183 ± 136 %ΔP_PA _× min. The combination of sub-threshold 8MM-IBMX with iloprost again enhanced the maximum P_PA _and PVR decrease in response to iloprost aerosolization and prolonged the vasodilatation to 50 min, with AUC values ranging at 1206 ± 177 %ΔP_PA _× min. No further amplification of the iloprost induced vasodilation was achieved by combination with sub-threshold doses of sildenafil plus 8MM-IBMX with an AUC of 1268 ± 115 %ΔP_PA _× min and an amplification of the iloprost effect on P_PA _decrease of 56.4 ± 4.0% (Fig. 2b). In contrast, further amplification was noted for the combination of inhaled iloprost with sub-threshold doses of sildenafil plus motapizone, which enhanced the P_PA _drop to 54.6 ± 3.7%, the PVR drop to 35% and increased the AUC to 1993 ± 166 %ΔP_PA _× min. In none of the groups, any significant decrease in systemic arterial pressure (Fig. 4) or deterioration of gas exchange (Table 1) was noted.

## Discussion

The inhalation of nebulized prostanoids for treatment of pulmonary hypertension is an approach targeting selective vasodilatation in well ventilated and aerosol accessible lung regions. This strategy has been developed to circumvent side effects of the conventional intravenous therapy with prostacyclin, e.g. systemic hypotension and ventilation-perfusion mismatch. As anticipated from previous studies in patients with severe pulmonary hypertension [4,5], aerosolization of iloprost was, indeed, effective in causing lung vasorelaxation without any decrease in systemic arterial pressure and any deterioration of gas exchange in the present rabbit model. After completion of the aerosolization maneuver, the iloprost effect levelled off within 20 – 30 min, which is somewhat more rapid than in the human system (45–90 min). This difference is most likely due to some species variance in the kinetics of the iloprost catabolic pathway: being chemically stable – in contrast to prostacyclin – iloprost is converted to dinor-and tetranor-iloprost metabolites via beta-oxidation [25]. The liver is known to be a major site of this catabolic pathway, however, recent studies in isolated perfused rabbit lungs demonstrated that the conversion of iloprost to these beta-oxidation products also takes place in the lung tissue itself [26].

Phosphodiesterases represent the major route for cAMP and cGMP degradation in cells, thereby limiting the downstream effects of adenylate and guanylate cyclase activating agents such as prostacyclin or NO. Until now, 11 types of PDEs have been characterized, which differ in substrate specificity and regulatory properties [9,27,28]. Within the lung, PDE 3 and 4 represent the major cAMP hydrolyzing pathways [10], and monoselective inhibitors of PDE 3 and PDE 4 have been shown to possess pulmonary vasodilatory potency [8,11,29].

Motapizone is a highly selective PDE3 inhibitor, with an IC_50 _value of 30 nM [30]. Thus, the finding that motapizone infusion causes dose-dependent pulmonary vasodilatation in intact rabbits with elevated pulmonary artery pressure is well in line with previous observations in this field. Notably, the motapizone effect was not pulmonary selective: in the dose range from 10 to 1000 μg/kg × min, both the pulmonary artery and the systemic artery pressure declined in a parallel fashion.

PDE1 gene products are expressed in cardiac tissues from several species [31,32]. A role of increased PDE1C expression in the cardio-protective effect of the stable prostacyclin derivative, 7-oxo-prostacyclin, indicates that PDE1C variants may be involved in tissue responses to cardiovascular stress [33]. In vascular smooth muscle cells derived from different species, several reports demonstrated PDE1 expression. PDE1C was shown to be present in proliferating smooth muscle cells [31,34] and an increased expression of PDE1A1 in rat aorta was shown to contribute to the development of nitroglycerin tolerance [35].

Concerning the lung vasculature, only very limited data on PDE1 expression is available [11]. Our group recently observed that PDE1C is strongly upregulated in the pulmonary artery media of human lungs with severe pulmonary hypertension (R. Schermuly et al., non-published results). It is in line with this notion that the selective PDE1 inhibitor 8MM-IBMX induced dose-dependent pulmonary vasodilation in the presently investigated acute pulmonary hypertension model, however, without being specific for the pulmonary circulation, as evident from the parallel decline of systemic arterial pressure.

The cGMP-specific phosphodiesterase PDE 5 is abundantly distributed in the lung tissue [13,14,36]. In a hypoxia-induced model of pulmonary hypertension in the rat, Cohen et al. demonstrated that the PDE 5 inhibitor E4021 selectively vasodilates the pulmonary circulation when being applied intravenously [36], and this observation was confirmed in a model of newborn lambs with persistent pulmonary hypertension [37]. Another specific PDE 5 inhibitor, E4010, has been shown to be a selective pulmonary vasodilatator in a hypoxic rat model of pulmonary hypertension [38]. Accordingly, the PDE 1/5/6 inhibitor sildenafil, which is approved for treatment of erectile dysfunction, was also recently shown to cause preferential pulmonary vasodilatation even when being systemically administered [39]. These data are well in line with the current finding in pulmonary hypertensive rabbits that intravenously infused sildenafil causes a doses-dependent decrease in pulmonary artery pressure, virtually without any decline in systemic arterial pressure.

The rationale to combine cAMP-elevating agents, like prostacyclin or the stable prostacyclin analogue iloprost, with PDE3 inhibitors is obvious, and studies from our group already showed a marked amplification and prolongation of the pulmonary vasodilatory response to aerosolized prostacyclin in the presence of type 3 PDE inhibitors [8,29]. The present investigation demonstrates that such synergistic effect also hold true for the PDE3 inhibitor motapizone and the longer acting agent iloprost: in the presence of minute doses of intravenously applied motapizone, the area under the curve of P_PA _decrease in response to the standard inhaled iloprost dose was nearly threefold increased, again without any decline in systemic arterial pressure or any deterioration of gas exchange being detectable.

**Figure 6:**
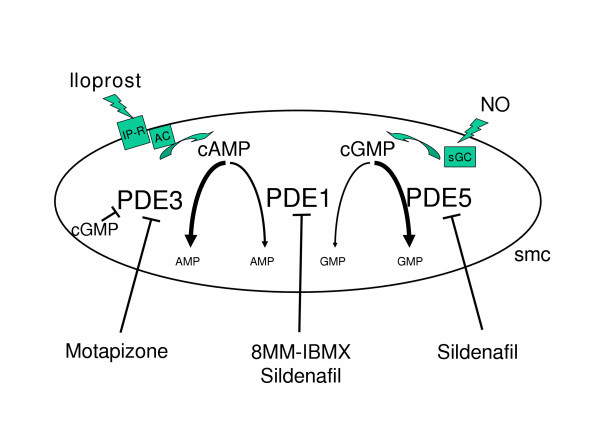
Schematic depiction of the crosstalk between different phosphodiesterases (PDE) and the effects of PDE inhibitors on cGMP and cAMP signaling. Different agonists, e.g. prostanoids or nitric oxide (NO) increase the intracellular concentrations of the second messengers cyclic adenosine monophosphate (cAMP) and guanosine monophosphate (cGMP). Phosphodiesterase (PDE) inhibitors stabilize the second messengers and amplify the efficacy of the agonists. 8MM-IBMX selectively blocks PDE1 which can hydrolyze both cyclic nucleotides. Motapizone inhibits PDE3, which hydrolyzes cAMP and is inhibitable by cGMP. Sildenafil blocks PDE5 and PDE1 and can therefore influence the cAMP and cGMP pathway. IP-R, prostacyclin receptor; AC, adenylate cyclase; sGC, soluble guanylate cyclase; smc, smooth muscle cell; PDE, phosphodiesterase.

Interestingly, similar potency to increase the response to inhaled iloprost was also noted for subthreshold doses of the PDE 1 inhibitor 8MM-IBMX. This might be anticipated to some extent, as PDE1 causes degradation of both cAMP and cGMP (Fig. 6), thereby directly effecting downstream signalling of iloprost, and indirectly modifying this pathway via cGMP sensitive PDE inhibitors. The fact, however, that the area under the curve of PPA reduction was similarly augmented as in the presence of motapizone (~threefold) suggests that PDE1 inhibitors are worth to be taken into consideration for further strategies to enhance beneficial prostanoid effects in the pulmonary circulation.

Furthermore, the current study demonstrates that the PDE 1/5/6 inhibitor sildenafil also amplifies the pulmonary vasodilatory response to inhaled iloprost, and that in this respect subthreshold systemic doses of sildenafil are again virtually as effective as subthreshold doses of the PDE3 inhibitor motapizone. This amplification of the iloprost-induced P_PA _decrease again occurred without any decline in systemic arterial pressure and any gas exchange disturbances. The mechanisms underlying this sildenafil effect deserve further elucidation. Given the pharmacological profile of this agent [40,41], it is unlikely that sildenafil caused relevant direct inhibition of lung PDE 3 and 4, thereby promoting prolongation of the half life of cAMP. Its effect may, however, well be explained by the known cross talk between the cAMP and the cGMP pathways: PDE 3 is inhibited by intracellular cGMP with an IC_50 _of 0.1–1 μM [42]. A significant inhibition of PDE 5 by sildenafil may thus result in cGMP accumulation, given the fact that there is some permanent baseline stimulation of this pathway via endogenous NO, and next to causing *per se *some vasodilatory effect, the sildenafil-induced cGMP may particularly be effective via PDE 3 inhibition and thereby enhanced sensitivity to inhaled iloprost. In addition to such mode of action, supported by previous studies in the cooperativity between the cGMP and the cAMP axis in the lung vasculature [8,43], further (non-cGMP related) effects of sildenafil in the pulmonary circulation may involve inhibition of PDE1 (Fig. 5). This view is supported by the above discussed efficacy of selective PDE1 inhibition by 8-MM-IBMX. The IC_50 _of sildenafil against PDE1 is about 280 nM [15], and although plasma levels of sildenafil are not addressed in this study, these levels could be achieved after sildenafil application.

Thus, both indirect inhibition of PDE3 by increased cGMP and direct inhibition of PDE1 may explain the effects of sildenafil on the iloprost-induced vasodilation. Our studies in co-application of sildenafil with motapizone on the one hand and 8-MM-IBMX on the other hand do, however, favour the sildenafil-PDE1 axis: whereas the combination of sildenafil with the PDE1 inhibitor 8MM-IBMX did not further amplify the iloprost-induced vasodilation over the effect of each agent alone, the combination of sildenafil plus motapizone effected a further strong amplification of the prostanoid induced vasodilation. Besides being of interest as to the mode of action of sildenafil, this finding suggests that an optimum strategy to combine PDE inhibitors may result in even further augmentation of pulmonary vascular prostanoid efficacy as compared to the choice of one selective PDE inhibitor as partner for the prostanoid. In conclusion, intravenous administration of the PDE1 inhibitor 8MM-IBMX, the PDE 3 inhibitor motapizone and the PDE 1/5/6 inhibitor sildenafil causes dose-dependent pulmonary vasodilation in a rabbit model of pulmonary hypertension, with sildenafil possessing selectivity for the lung vasculature. Most interestingly, when applied in subthreshold doses, all PDE inhibitors enhanced and markedly prolonged the vasodilatory response to inhaled iloprost, without any systemic pressure decline or deterioration of gas exchange being detectable. Maximum efficacy was noted upon combination of sildenafil and motapizone. Combination of low dose systemic PDE inhibitors might thus be considered for enhancement and in particular prolongation of the lung vasorelaxant response to inhaled iloprost.

## Abbreviations

P_PA_, mean pulmonary artery pressure; P_SA_, mean aortic pressure; CO, cardiac output; U46619, stable thromboxane-A_2 _analogue; PDE, phosphodiesterase; Mota, motapizone; Sil, sildenafil; Ilo, iloprost.
